# Leaf litter breakdown along an elevational gradient in Australian alpine streams

**DOI:** 10.1002/ece3.9433

**Published:** 2022-10-18

**Authors:** Lloyd P. Werry, Mirco Bundschuh, Simon M. Mitrovic, Richard P. Lim, Ben J. Kefford

**Affiliations:** ^1^ School of Natural Resources PNG University of Natural Resources and Environment Kokopo Papua New Guinea; ^2^ School of Life Sciences, Faculty of Science University of Technology Sydney Sydney New South Wales Australia; ^3^ iES Landau, Institute for Environmental Sciences Universität Koblenz‐Landau Landau Germany; ^4^ Department of Aquatic Sciences and Assessment Swedish University of Agricultural Sciences Uppsala Sweden; ^5^ Centre for Applied Water Science, Institute of Applied Ecology University of Canberra Canberra Australian Capital Territory Australia

**Keywords:** litter, macroinvertebrates, organic matter, water temperature

## Abstract

The breakdown of allochthonous organic matter, is a central step in nutrient cycling in stream ecosystems. There is concern that increased temperatures from climate change will alter the breakdown rate of organic matter, with important consequences for the ecosystem functioning of alpine streams. This study investigated the rate of leaf litter breakdown and how temperature and other factors such as microbial and invertebrate activities influenced this over elevational and temporal gradients. Dried leaves of Snow Gum (*Eucalyptus pauciflora*) and cotton strips were deployed in coarse (6 mm), and fine (50 μm) mesh size bags along an 820 m elevation gradient. Loss of mass in leaf litter and cotton tensile strength per day (*k* per day), fungal biomass measured as ergosterol concentration, invertebrate colonization of leaf litter, and benthic organic matter (mass and composition) were determined. Both microbial and macroinvertebrate activities were equally important in leaf litter breakdown with the abundance of shredder invertebrate taxa. The overall leaf litter breakdown rate and loss of tensile strength in cotton strips (both *k* per day) were greater during warmer deployment periods and at lower elevations, with significant positive relationships between mean water temperature and leaf breakdown and loss of tensile strength rate, but no differences between sites, after accounting for the effects of temperature. Despite considerably lower amounts of benthic organic matter in streams above the tree line relative to those below, shredders were present in coarse mesh bags at all sites. Ergosterol concentration was greater on leaves in coarse mesh bags than in fine mesh bags, implying differences in the microbial communities. The importance of water temperatures on the rate of leaf litter breakdown suggests the potential effects of climate change‐induced temperature increases on ecological processes in such streams.

## Graphical abstract

We aimed at understanding how spatial and temporal water temperatures influenced AOM breakdown in alpine streams. Microbial and macroinvertebrate activities were monitored and showed to be elevated at lower elevations and during warmer periods. Climate change induced warming will therefore potentially change alpine stream ecosystem, standing biomass and promote increased nutrient cycling through AOM breakdown.

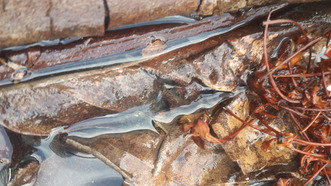

## 1 INTRODUCTION

Allochthonous organic matter is an important source of energy for lotic environments (e.g., Bo et al., 2014; Tank et al., 2010; Woodward et al., 2012), and is a central component of ecological theories such as the River Continuum Concept (Vannote et al., 1980) and the River Wave Concept (Humphries et al., 2014). Leaf litter in stream ecosystems represents a significant proportion (>60%) of the total allochthonous organic input as compared with other parts of terrestrial plants (e.g., bark, branches, and flowers; Gessner & Chauvet, 2002; Young et al., 2008), which all complement the involvement of benthic algae and diatoms in the autochthonous production. There, leaf litter undergoes a sequence of processes from leaching, microbial colonization, and finally, its breakdown due to both physical abrasion and biological activity (Bo et al., 2014; Tank et al., 2010).

Benthic microbes and macroinvertebrates are considered central in the breakdown of organic matter (Hieber & Gessner, 2002). Macroinvertebrate shredders, a common functional feeding group in streams with high allochthonous input (Merritt et al., 2002; Vannote et al., 1980), feed on the organic matter after microbial conditioning, with the latter being characterized by an enrichment of lipids, proteins, and nutrients as well as softening of leaf structures. Breakdown efficiency of leaf litter is influenced by multiple factors; the leaf species and their intrinsic properties (Benfield et al., 2017; Gessner & Chauvet, 1994; Lecerf & Chauvet, 2008); water chemistry (including nutrients and salinity; Zhai et al., 2021); water temperature (Taylor & Andrushchenko, 2014); elevation (Salinas et al., 2011); stream substrate (Hoover et al., 2006; Suberkropp & Chauvet, 1995); and local macroinvertebrate community composition (Bo et al., 2014). Nutrients, up to a threshold concentration, stimulate microbial activity, making leaf litter more palatable for macroinvertebrates, ultimately stimulating its breakdown (Woodward et al., 2012). This nutrient‐stimulated breakdown becomes more profound in streams below the tree line compared with that above the tree line, where increased allochthonous and autochthonous sources are complementary (Dobson & Hildrew, 1992; Manning et al., 2021; Tilman, 1982). This difference in energy inputs (autochthonous vs. allochthonous) can drive the composition and distribution of macroinvertebrates at the catchment scale (Ligeiro et al., 2010; Vannote et al., 1980; Ward, 1992). Thus, assessing relationships between macroinvertebrate community composition and availability of food resources are important in understanding carbon and nutrient processing within a stream food web (Bo et al., 2014).

No information exists on the role of macroinvertebrates in ecosystems functions such as leaf litter breakdown in Australian sub‐alpine and alpine streams despite this area being one of the six eco‐regions highly vulnerable to climate change (Hennessy et al., 2007; Pickering, 2007) and providing critical ecosystem services for humans such as drinking and irrigation water for inland (arid and semi‐arid) Australia and hydroelectricity generation. One hundred and fourteen taxa comprising mostly mayflies, stoneflies, and caddis flies have been recorded in streams of the upper alpine reaches of the Snowy Mountains (Suter et al., 2002). Australian mountains have no extant glaciers or year‐round snow packs. Consequently, alpine organisms generally have nowhere to migrate up to as climate change alters their habitat. Thus, climate change may represent a major threat to the biodiversity and ecological function of sub‐alpine and alpine Australian macroinvertebrate communities and the ecosystem functions and services they provide, which includes the breakdown of organic matter.

This study investigated the rate of leaf litter breakdown and how temperature and other factors such as microbial and invertebrate activities influenced this over elevational and seasonal gradients. We hypothesized that litter breakdown increases with increasing temperature associated with both seasonal and elevational changes. Having the tree line as a divide in terms of the supply of allochthonous organic matter into streams, we also hypothesized that streams and sites below the tree line have more leaf litter than those above it, and that, consequently, the biota present at sites below the tree line is more amenable to using terrestrial leaves as a food source, so that leaf litter breakdown is greater below the tree line than above it. We further determined what macroinvertebrates were associated with leaf litter and thus influencing their breakdown. Importantly, our sampling design included sampling across seasons and along an elevational gradient that was not confounded with stream or catchment size. This study ultimately provides information on the risks of climate–change–induced temperature increases on leaf litter breakdown.

## 2 MATERIALS AND METHODS

### 2.1 Study area and sites

**FIGURE 1:**
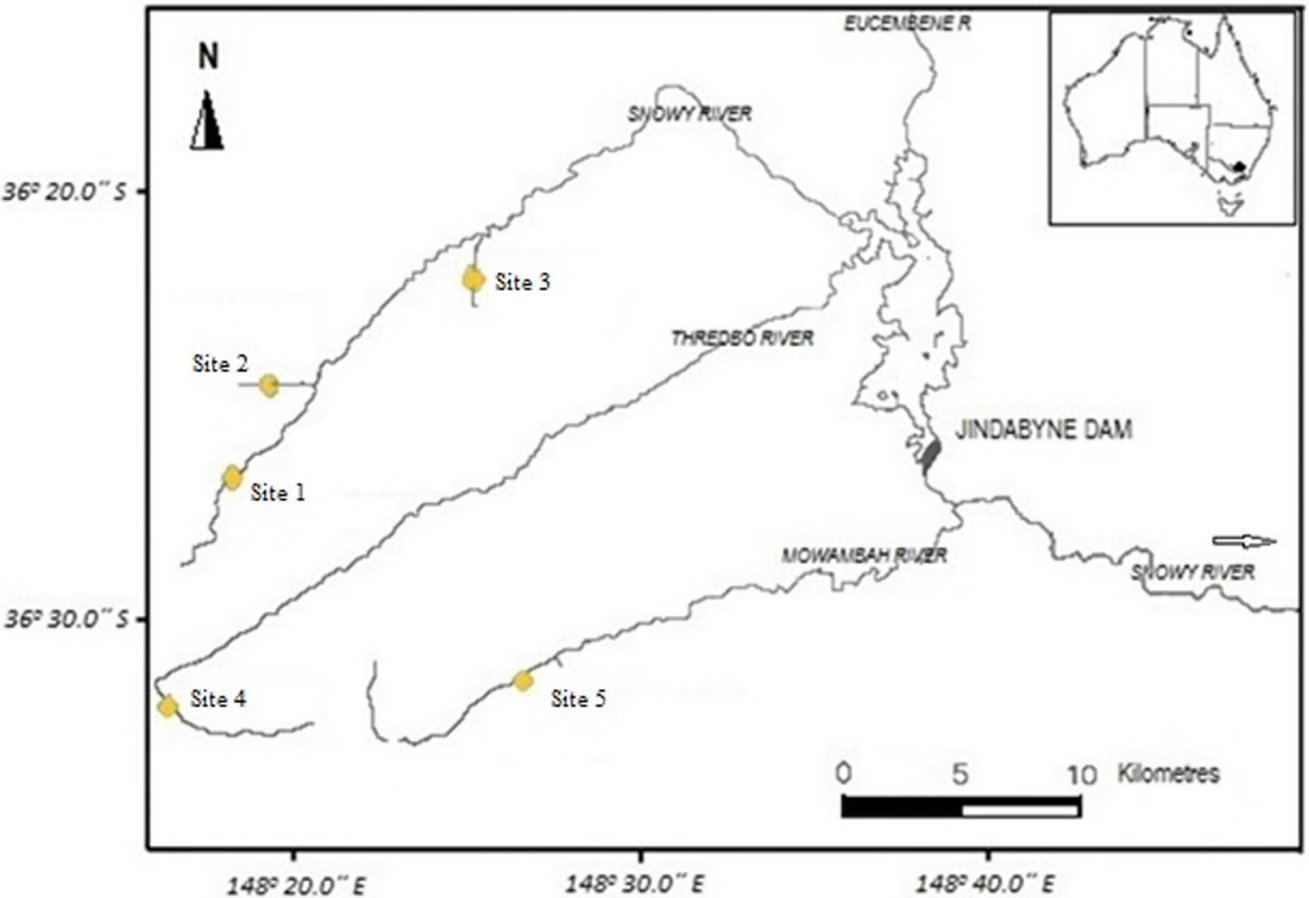
Study sites on the selected alpine streams (Site 1—Snowy River, Site 2—Club Lake Creek Site 3—Diggers Creek, Site 4—Thredbo River, and Site 5—Mowamba River).

Five (5) headwater streams in the upper Snowy River catchment were selected along an elevation gradient (Figure 1). Sites were selected in a series of tributaries, so that elevation was not confounded with stream size or catchment size (as per Gill et al., 2016; Shah et al., 2015, 2017). All stream sites were thus of a similar size (~4.5 m stream width), had riffle/run habitats, and were along an elevational gradient at ~200 m intervals. The substrate profile was similar. All sites are located either within or near the border of the Kosciusko National Park area with low human activity (Table 1).

**TABLE 1:** Description of stream sites

Name of stream	Elevation (m [asl])	Latitude and longitude	Average stream size width ± SE (depth ± SE) m	Vegetation cover and site description
Site 1: Snowy River	1935	36°27′48.34″S; 148°17′28.02″E	6.0 ± 0.25 (0.1 ± 0.012)	Above tree line (alpine). Exposed to high winds, snow cover in winter‐early spring months No vegetation cover. Site has a decline of approximately <5° with a 15% riffle/run. Lowest depth and flow
Site 2: Club Lake Creek	1744	36°27′485.35″S; 148°17′47.47″E	5.6 ± 0.15 (0.15 ± 0.027)	Above tree line (alpine). Exposed to high winds, snow cover in winter‐early spring No vegetation cover, a decline of <10°, and 50% riffle/run
Site 3: Diggers Creek	1415	36°20′08.11″S; 148°29′21.26″E	2.5 ± 0.17 (0.16 ± 0.08)	Below tree line (sub‐alpine). Dense shrubs (80%–90%) and gum cover (50%), <15° decline and 70% riffle/run
Site 4: Thredbo River	1556	36°26′33.72″; 148°18′02.16″E	3.7 ± 0.23 (0.28 ± 0.013)	Below tree line (sub‐alpine). Sparsely covered with low shrubs (10%–15% cover), a <10° decline, and 40% riffle/run
Site 5: Mowambah River	1110	36°30′04.53″S; 148°28′53.36″E	3.6 ± 0.33 (0.26 ± 0.019)	Below tree line (sub‐alpine). Vegetation and tree canopy (50%–60% cover) and denser cover further upstream. Approximately <10° decline and 20% riffle/run

The local topography varied with bog and fen at the top of the catchment and gum forests along steep topography lower in the catchment. The vegetation was dominated by gum (*Eucalyptus*) up to the alpine zone marked by Snow Gum (*E. pauciflora*), which defines the tree line (Costin, 1989). At lower elevations, the riparian vegetation is normally shaded by Alpine Ash (*E. delegatensis*), *E. cypellocarpa*, and *E. dives*, with undergrowth of shrubs, mainly the mountain tea tree (*Leptospermum grandifolium*) along Diggers Creek and Mowamba River and Alpine Baeckea (*Baeckea gunniana* Shauer) along the Thredbo River. Above the tree line, *B. gunnianna* appears sparingly along Club Lake Creek among a mosaic of heath, grass, and alpine herbs (Costin et al., 2000).

In the stream channel, exposure and protrusion of bedrock are prominent (10%–20%). Sites above the tree line (Snowy River and Club Lake Creek) are interspersed with 15%–20% of large boulders, and more than 50% of substrate across streams is comprised of cobbles. Riparian trees are common at sites below the tree line with shading by the vegetation of >4 m height at Mowamba River, Diggers Creek, and Thredbo River sites (Table 1). Aquatic macrophytes were not observed at any of the sites.

### 2.2 Water quality measurements and sampling

Electrical conductivity, pH, and dissolved oxygen were measured in situ at each site during all sampling episodes using a calibrated WTW multiprobe meter for pH (SenTix 41, pH 330, TetraCon 325, Cond330i, OxiCal‐SL probe, Oximeter 330; Weilheim). Hourly water temperature was measured using Hobo Pendant (Onset).

Instantaneous mean flow rate (m/s) was estimated using a calibrated hydroprop from seven replicated measurements (as per Marchant et al., 2011; Wahizatul et al., 2011). Stream discharge (m^3^/s) was calculated using the approximate average cross‐sectional area of the stream reach and its average velocity over the reach. Additionally, 50 ml samples of filtered water (Minisart sterile filters, 0.45 μm pore size) were collected for nutrient analysis. Sample bottles were pre‐rinsed with the filtered water before samples were taken. Samples were kept frozen until analysis for total oxidized nitrogen and phosphorus using a segmented flow analyzer (Model FS3100, ALPKEM) with detection limits at 0.002 mg/L for measured nutrients.

### 2.3 Preparation of leaf bags

Locally common snow‐gum (*Eucalyptus pauciflora*) leaves were collected prior to abscission from Thredbo River at Dead Horse Gap (36°31′22″S, 148°15′51″E) 4 weeks before each deployment. In the laboratory, leaves were oven‐dried (48 h at 60°C) and stored frozen (−18°C) until leaf bag preparation. Approximately 4.5 g (0.001 g precision) of dried leaves were placed into coarse polyethylene mesh bags (mesh size: 6 mm; cylindrical length: 15 cm × 25 cm) or fine nylon bags (mesh size: 50 μm; cylindrical length: 10 cm × 20 cm); with the fine mesh bag placed inside each coarse mesh bag. Leaves in the fine mesh bags were only accessible to microbes, while the coarse mesh bags were accessible to both microorganisms and invertebrates; the fine mesh bags served as means of measuring leaching and microbial activity (Boulton & Boon, 1991).

A set of six coarse mesh bags with fine mesh bags therein was deployed at each site from March to April and April to May of 2013. Half of the coarse mesh bags were retrieved after 28 and the remaining at 56 days. In late May 2013, another six bags per type were deployed and retrieved after 150 days (October). Ten bags of each were again deployed for 56 days between December 2013 and January 2014; and a further five bags of each type for 56 days (late February until early April 2014). All bags were placed in riffle‐run locations and anchored to the bottom of the stream bed either onto submerged woody roots of riparian vegetation or to metal rods driven into the substrate. Four additional coarse and fine mesh bags were immersed in river water and retrieved immediately, and taken to the laboratory to account for mass losses due to handling, serving as field blanks. No measurement was taken for losses due to physical abrasion, which is considered a minor influence on leaf weight loss (Imberger et al., 2008). After retrieval, all leaves were stored at −18°C prior to freeze‐drying. The freeze‐dried weights were obtained for calculating leaf mass loss.

Leaf mass losses were obtained after measuring their dry weights (precision 0.01 g) before and after stream exposure. These losses were corrected by the field blanks; which were briefly dipped in the streams, and their dry weights measured. Differences in leaf mass loss between coarse and fine mesh bags were calculated and reflect the activity of macroinvertebrates.

For the microbial degradation of cotton strips, unbleached standard cotton (EMPA) was cut into 3 × 10 cm strips and autoclaved for 1 h at 120°C (Schäfer et al., 2012). A cotton strip was then placed in each of the coarse and fine mesh bags (with the leaves). Immediately upon retrieval, cotton strips were cleaned and soaked in 70% ethanol for 10 min, air‐dried, and stored at −18°C. This inhibited further microbial degradation during storage. In order to account for any impact of handling, four cotton strips served as field blanks.

From the centre of the cotton strips, 2 cm × 5 cm pieces were cut and used to measure their tensile strengths (e.g., Wang et al., 2008). Using a tensiometer (an Instron® 4500 Universal Testing Systems with a testing frame 5500R electronics), strips were clamped at both ends and stretched to breaking‐point at 3 mm/s. Readings of load and stretch lengths were obtained via Bluehill 2 software program (www.instron.com.au).

### 2.4 Ergosterol analysis

Ergosterol concentration was determined as a proxy for fungal biomass on leaves as fungi are regarded as critical to the microbial breakdown of leaf litter in streams and conditioning of leaves for breakdown by invertebrate shredders. Freeze‐dried and ground leaf litter sub‐samples (100 mg each) collected from the bags were assayed for ergosterol as described by Gessner and Schmitt (1996). Briefly, ergosterol was extracted from leaf samples for half an hour in 10 ml of alkaline methanol at 80°C. After cooling down to room temperature, the ergosterol was purified by solid phase extraction (Sep‐Pak Vac RC tC18 500 mg sorbent, Waters). Separation of ergosterol was done on a high‐performance liquid chromatograph (Agilent Technologies, 1200 Series) and measured with an ultraviolet detector (wavelength of 282 nm). Using a standard calibration curve prepared with the chemical standard (Fluka, purity at 97%), ergosterol concentrations were determined and normalized to the dry mass of the leaves (Gessner & Schmitt, 1996).

### 2.5 Particulate organic matter content of the benthos

Particulate organic materials were sampled using seven replicate Surber samples per sampling episode to provide estimates of the quality and types of organic matter available in the benthos. Organic materials were collected and separated from macroinvertebrates in the Surber samples. The composition of organic matter was broadly categorized as: Aquatic plants (for algae and moss) and terrestrial grass (for grasses but also all other monocots), small leaves (from shrubs), large leaves (mainly from *Eucalyptus*), and twigs/bark/wood (woody plants). The dried samples were weighed in crucibles and incinerated in a muffle oven for 5 h at 550°C to determine ash weights. The ash content of these samples was determined according to the proportion of the types of organic matter and normalized to g/m^2^ of the sampled area.

### 2.6 Macroinvertebrate colonization of leaf bags

Following the collection of leaf bags, macroinvertebrates in the bags were carefully removed and stored in 70% ethanol. Seven samples in the vicinity of the leaf bags were collected with a Surber sampler, and the contents were preserved in 70% ethanol for sorting in the laboratory. The sorted macroinvertebrates from the coarse mesh bags and Surber sampler were identified to the family level and enumerated. Macroinvertebrates from leaf bags were expressed in percentage composition to compare with that of the Surber samples.

### 2.7 Data handling and analysis

For leaf mass loss and cotton tensile strength loss, means (+SE) were calculated for all sites and deployments. The breakdown rate *k* for leaf litter mass loss and cotton tensile strength loss for each site *i* per day was determined with modification to the equation of Schäfer et al. (2012). The mass loss rate *k*(*i*) was obtained by taking a negative natural logarithm (−ln) of a quotient of mass lost (*Si[t]*) over the original mass (*Si[0]*) in a period of exposure *t*.

$$k\;\left(i\right)=\frac{-\ln\left(\frac{\mathrm{Si}\left(t\right)}{\mathrm{Si}\left(0\right)}\right)}{t}$$

Leaf mass loss was expressed as percentage loss per day (*k* per day). Similarly, the percentage tensile loss per degree day was calculated for cotton strips. Analysis of variance (ANOVA) was carried out on leaf mass loss and the rate of cotton tensile strength loss against sites and sampling episodes. Analysis of covariance (ANCOVA) was also conducted with the site as a fixed factor and mean temperature over the deployment period of the leaves or cotton strips as a continuous variable.

Macroinvertebrate data of both leaf bags and respective benthic samples were analyzed using Primer V6 (Primer – E Ltd, Plymouth), a multivariate statistical software using Bray–Curtis index to calculate a similarity between all samples (Clark & Warwick, 2001).

Differences between spatial and temporal patterns in macroinvertebrate assemblages and distribution were tested using permutational multivariate analysis of variance (Andersons, 2001). This was performed to determine if the macroinvertebrate assemblages differed between the coarse mesh bag and benthic communities, deployment episodes, and sites. The data were square‐root transformed, and Bray–Curtis distance was used to form a similarity matrix. Multi‐dimensional scaling (nMDS) plots were produced to illustrate potential differences among sites and deployments. The stress level (0.24) of the nMDS plot indicated an acceptable “goodness of fit” of the data as plotted in two dimensions (Clark & Warwick, 2001). The similarity, of Percentages (SIMPER) analysis was then performed on the clustering of data observed on the nMDS plots. The SIMPER analysis was performed to identify the main macroinvertebrate families driving the clustering among sites and sampling episodes.

Ash free dry mass of organic matter collected from the benthos was grouped into the type of organic matter (g/m^2^). The data were then tested for differences between sites and sampling episodes using ANOVA.

## 3 RESULTS

### 3.1 Physical and chemical characteristics of the sites

Total nitrogen was highest for the Snowy River site and roughly half this concentration at other sites. Phosphorus concentrations of the two sites above the tree line were <2.0 μg/L but between 3.8 and 14 μg/L at other sites. Electrical conductivity measured for sites below the tree line (i.e., Diggers Creek, Thredbo River, and Mowamba River sites) were 2–3 times higher than those above the tree line (Snowy River and Club Lake Creek sites). In all cases, electrical conductivity was below 32 μS/cm at 25°C. Mean and minimum temperatures decreased, but the maximum temperature increased, with increasing elevation (Table 2).

**TABLE 2:** Physico‐chemical properties of stream water was measured on five occasions between December 2012 and October 2013

Parameters	Snowy River		Club Lake Creek		Thredbo River		Diggers Creek		Mowamba River	
	Mean (±SE)	*n*	Mean (±SE)	*n*	Mean (±SE)	*n*	Mean (±SE)	*n*	Mean (±SE)	*n*
Total Nitrogen (μg/L as NO_2_‐N + NO_3_‐N + TKN‐N) Total N	260 (±27.7)	4	87.5 (±12.9)	4	93.8 (±9.8)	4	107 (±11.8)	4	138 (±31.4)	4
Phosphorus (μg/L as PO_4_‐P)	<2.0	3	<2.0	4	3.8 (±0.5)	4	4.0 (±0.6)	3	14.3 (±3.4)	3
Electrical conductivity (μS/cm @ 25°C)	5.3 (±1.15)	4	7.13 (±0.84)	4	11.4 (±0.64)	5	32.7 (±1.73)	4	32.3 (±1.91)	3
pH	6.6 (±0.15)	5	6.76 (±0.09)	4	6.86 (±0.22)	5	6.83 (±0.21)	4	6.81 (±0.23)	5
Temperature (°C)										
Mean	6.8 (±0.26)	289	6.7 (±0.22)	289	7.9 (±0.20)	286	8.6 (±0.18)	259	9.0 (±0.19)	291
Minimum	−1.7		−0.04		−0.03		0.67		0.33	
Maximum	28		26		25		21		25	
Degree‐days	66,092.84	289	57,008.62	289	156,462.3	286	32,029.05	259	60,782.63	291
Discharge (m^3^/s)	0.12 (±0.03)	5	0.08 (±0.03)	5	0.22 (±0.07)	5	0.07 (±0.01)	5	0.31 (±0.10)	5
Dissolved oxygen (mg/L)	10.7 (±0.6)	5	9.9 (±0.6)	4	9.6 (±0.5)	4	9.9 (±0.5)	5	9.3 (±0.5)	5
% Saturation	80.3	–	85.9		85.8		86.3		91.6	

*Note*: Mean temperatures were calculated from daily means for the whole period. The ranges for each site are given as minimum and maximum temperatures, as the lowest and highest temperatures recorded for the entire measurement period. Degrees days are presented for each site as covering the entire measurement period.

### 3.2 Leaf litter breakdown

**FIGURE 2:**
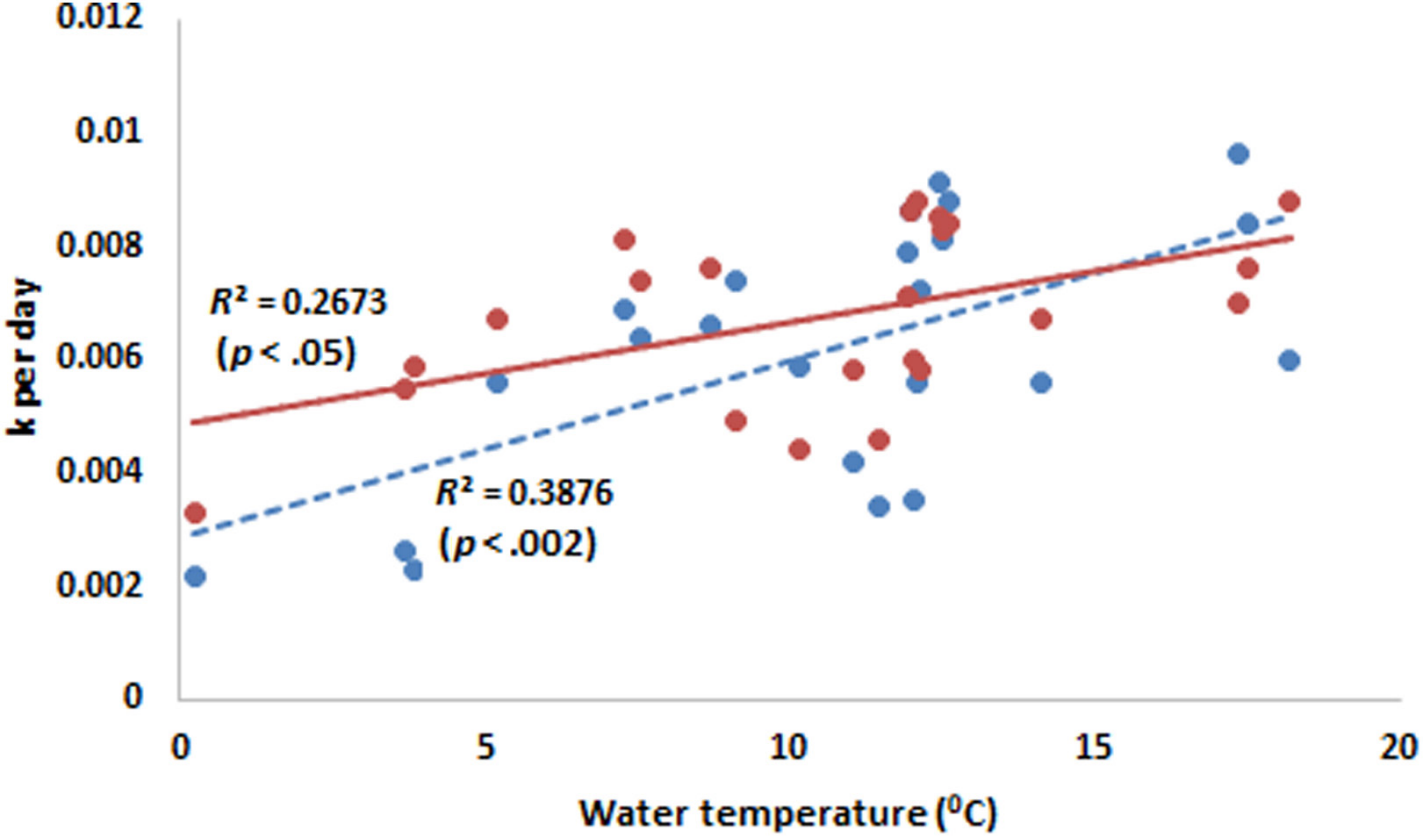
Relationships between daily mean temperature and mean rate of litter breakdown (*k* per day) at each site and deployment period between March 2013 and April 2014. *R* ^2^ and *p*‐values are from simple linear regression for the value of *k* per day being predicted from water temperature. Leaf litter in fine mesh (− −) and coarse mesh (¬‐) bags are represented.

Rates of leaf litter breakdown (*k* per day) were significantly different between sites (*F* = 4.81, *p* = .007) and between fine and coarse mesh bags (bag type; *F* = 9.19, *p* = .007) and showed a significant site by bag‐type interaction (*F* = 3.02, *p* = .042). The *k* values were significantly higher for leaves from coarse mesh bags than that from the fine mesh bags, showing that both macroinvertebrate and microbial activity were contributing to leaf breakdown (Figures 2 and S2). The site by mesh size interaction suggested that the difference between the two bag types was not consistent among the sites. The interaction was a result of a relatively small difference in the breakdown rates between the bag types at sites above the tree line (i.e., 1 and 2), relative to those sites below the tree line (i.e., 3, 4, and 5; Table 3). The *k* values for both fine and coarse mesh bags were lower during the cooler deployments (e.g., April–May 2013) than in the warmer deployments (November–December 2013; Table 3).

**TABLE 3:** Breakdown rates *k* per day (± standard error of the mean) for *E. pauciflora* leaf litter placed in fine (a) and coarse mesh bags (b) in streams in the Snowy Mountains (Snowy River, Club Lake Creek, Thredbo River, Diggers Creek, and Mowamba River) between March 2013 and April 2014.

Sampling episode	Processing coefficients				
	Snowy river	Club Lake Creek	Thredbo River	Diggers Creek	Mowamba River
	*k* per day	*k* per day	*k* per day	*k* per day	*k* per day
(a) Fine					
April–May 2013	0.0069 (0.0002)	0.0074 (0.0001)	0.0079 (0.0002)	0.0076 (0.0002)	0.0066 (0.0001)
June–Oct 2013	0.0022 (0.0001)	^a^	0.0023 (0.0001)	^a^	0.0026 (0.0001)
Nov–Dec 2013	0.0056 (0.0002)	0.0056 (0.0001)	0.0060 (0.0002)	0.0064 (0.0002)	0.0068 (0.0002)
Feb–Apr 2014	0.0034 (0.0001)	0.0032 (0.0002)	0.0032 (0.0001)	0.0029 (0.0001)	0.0035 (0.0001)
(b) Coarse					
April–May 2013	0.0081 (0.0003)	0.0074 (0.0002)	0.0139 (0.0003)	0.0087 (0.0003)	0.0072 (0.0003)
June–Oct 2013	0.0038 (0.0002	^a^	0.0059 (0.0002)	^a^	0.0065 (0.0002)
Nov–Dec 2013	0.0057 (0.0002)	0.0058 (0.0002	0.0088 (0.0002)	0.0076 (0.0002)	0.0070 (0.0003)
Feb–Apr 2014	0.0036 (0.0001)	0.0038 (0.0001)	0.0058 (0.0002)	0.0044 (0.0002)	0.0060 (0.0002)

^a^ From lost leaf bags.

In both bag‐types, higher percentages (ca. 50%–60%) of mass loss were observed for sites below the tree line relative to those above the tree line (ca. 10%–20%) and across all sites during warmer deployments. An increased contribution of macroinvertebrates to leaf litter mass loss was observed from April to May than at any other deployment periods (Table 3). No significant difference was observed when leaf litter mass loss was due primarily to macroinvertebrate activity alone (*R* ^2^ = 0.0582; *p* = .245), although it slightly decreased with increasing temperature (Figure S2).

There were no detectable differences in *k*‐values and temperature between the sites. However, ANCOVA showed a significant linear relationship between temperature and leaf litter breakdown rate (*k* per day), especially for fine mesh bags (*F* = 10, *p* = .006). While the relationship was not quite as strong in coarse mesh bags, it was significant (*F* = 5.8, *p* = .028; Figure 2). For both bag types, there was no evidence of any differences between the sites (Coarse *F* = 0.4, *p* = .8, Fine *F* = 0.2, *p* = .9).

#### 3.2.1 Fungal ergosterol as a proxy for microbial conditioning

The largest difference in ergosterol concentrations was between bag types (*F* = 33.6, *p* < .001), with coarse mesh bags having up to three‐fold higher concentrations (indicating greater fungal biomass) than their fine‐meshed counterparts. Significant differences in ergosterol concentrations were noted between sites (*F* = 2.6, *p* = .041) and between sampling episodes (*F* = 8.7, *p* < .001). Snowy River showed the lowest ergosterol concentrations in both bag‐types across sampling episodes. There were no significant interactions (*p* = .59–.91) between any of these factors (sampling episodes or elevation).

**FIGURE 3:**
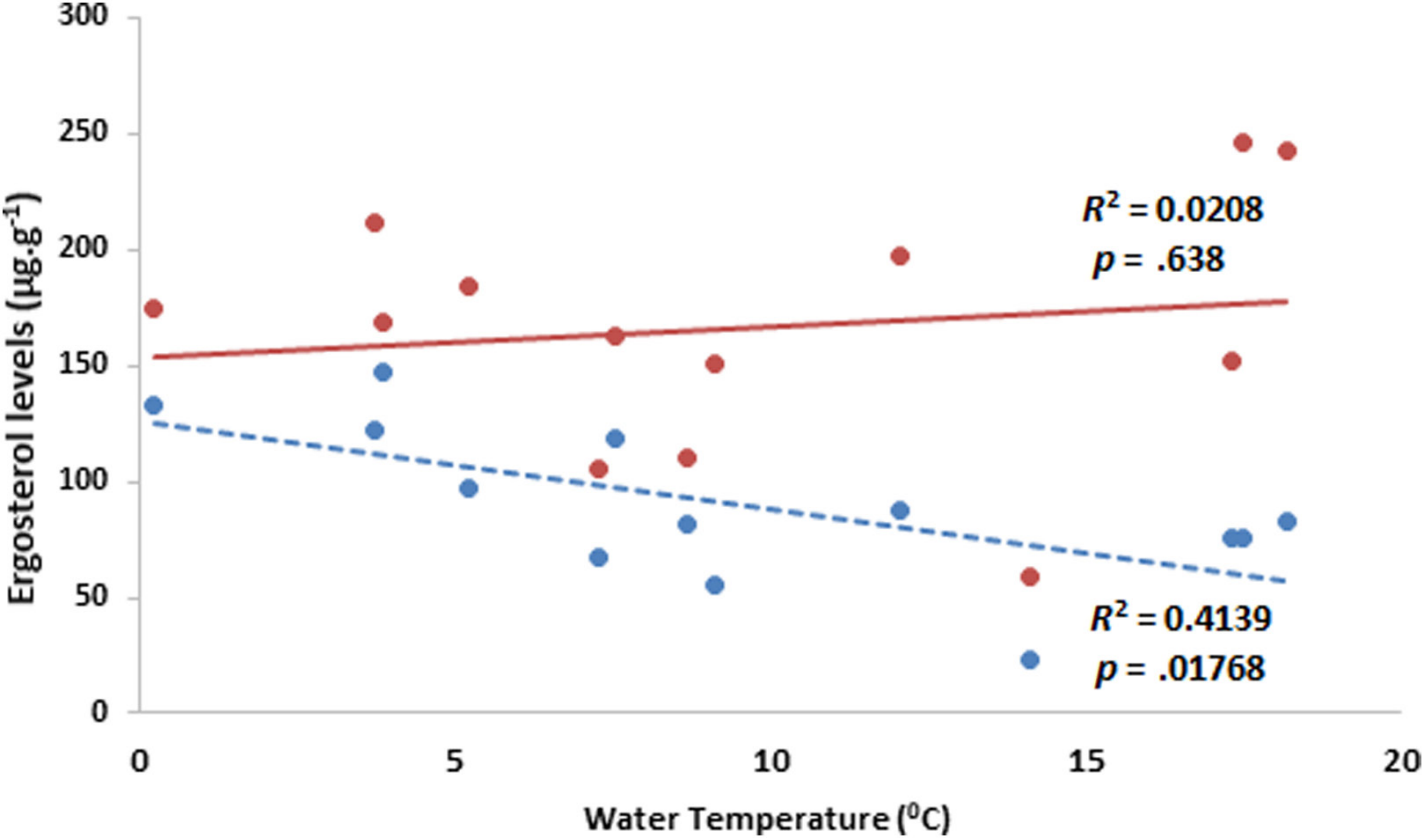
Relationship between daily mean water temperatures and mean ergosterol levels (μg/g) for all sites and deployment periods. *R* ^2^ and *p*‐values are from simple linear regression for the value of ergosterol levels being predicted from water temperatures. Ergosterol data‐points and solid trend line in red represent coarse mesh bags, and blue data‐points and dashed trend line are for fine mesh bags.

For differences in ergosterol levels in bag types, ANCOVA showed that there was a significant linear relationship between temperature and ergosterol levels (*F* = 9, *p* = .02) for fine mesh bags. However, there was no effect of temperature for coarse mesh bags (*F* = 0.8, *p* = .55; Figures 3 and S3). For both bag types, ANCOVA showed no difference between sites (Coarse: *F* = 1.56, *p* = .27; Fine: *F* = 0.8, *p* = .55).

#### 3.2.2 Cotton tensile loss

**FIGURE 4:**
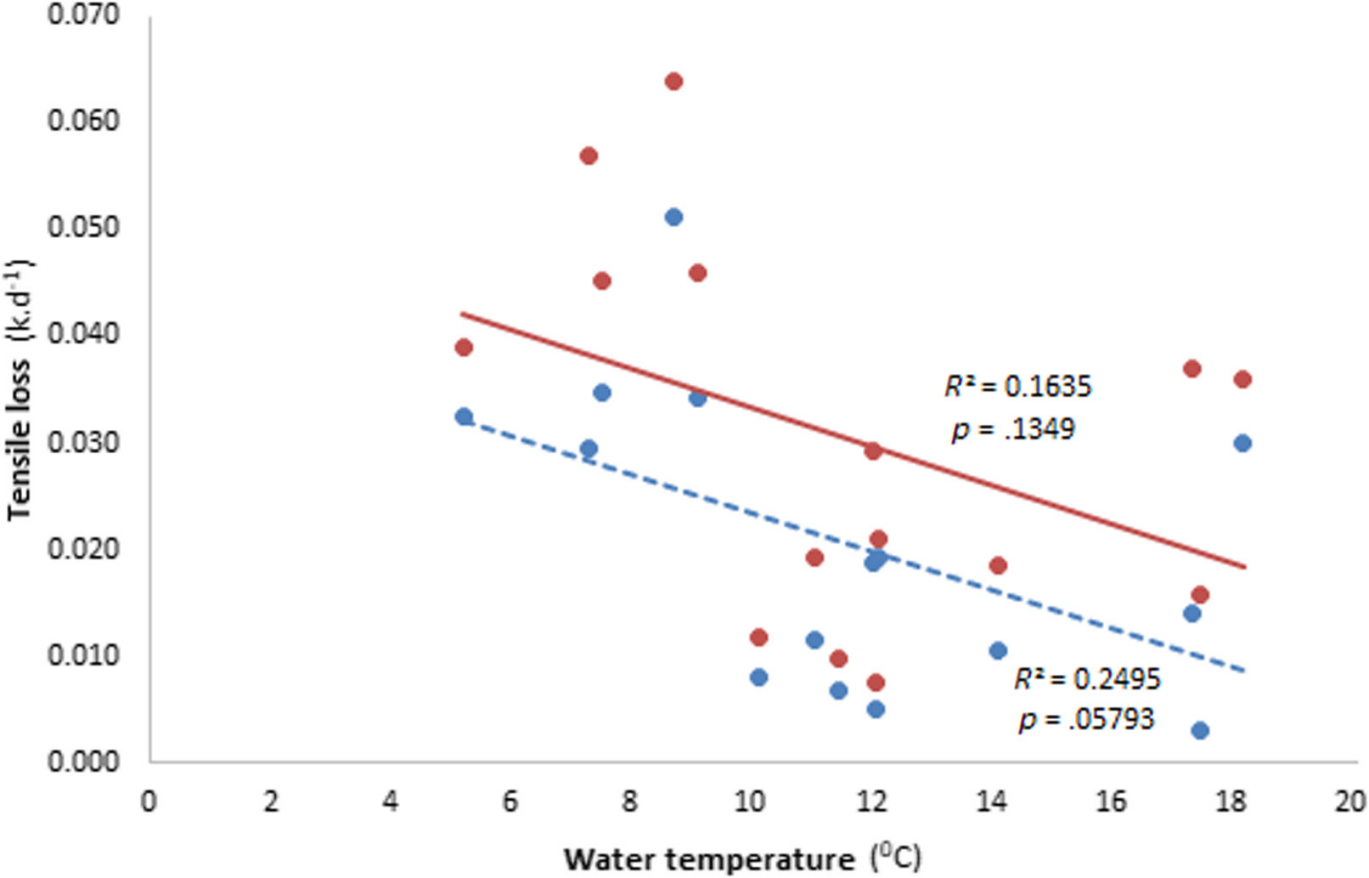
Relationship between daily mean water temperatures and mean cotton‐strip tensile loss (*k* per day) for all sites and deployment periods. *R* ^2^ and *p*‐values are from simple linear regression for the value of *k*‐values being predicted from water temperatures. Data‐points and solid trend line in red represent coarse mesh bags, and blue data‐points and dashed trend line are for fine mesh bags.

Testing for tensile loss between bag types, ANCOVA showed a linear relationship between tensile loss and temperature (Coarse: *F* = 5.3, *p* = .047; Fine: *F* = 10, *p* = .011). Tensile loss decreased in both bag types as temperature decreased, and these decreases were proportionate across sites (Figure 4). However, there were no significant differences between sites (Coarse: *F* = 1.5, *p* = .274; Fine: *F* = 2.2, *p* = .147). Both bag types showed negative relationships between the rate of tensile loss and water temperature (Coarse: *R* ^2^ = 0.16, *p* = 0.13; Fine: *R* ^2^ = 0.25, *p* = .057; Figure 4).

The cotton tensile loss (*k* per day) was significant (*F* = 3.08, *p* = .006) in a two‐way interaction between site and sampling episodes. The interaction reflected the highly variable *k*‐values observed for different sampling episodes and sites. For example, a high *k*‐value (0.064) was shown for coarse mesh bags at Mowamba River during a cooler deployment (April–May) and a low *k*‐value (0.0031) during a warmer deployment (November–December).

#### 3.2.3 Particulate organic content of benthos

**FIGURE 5:**
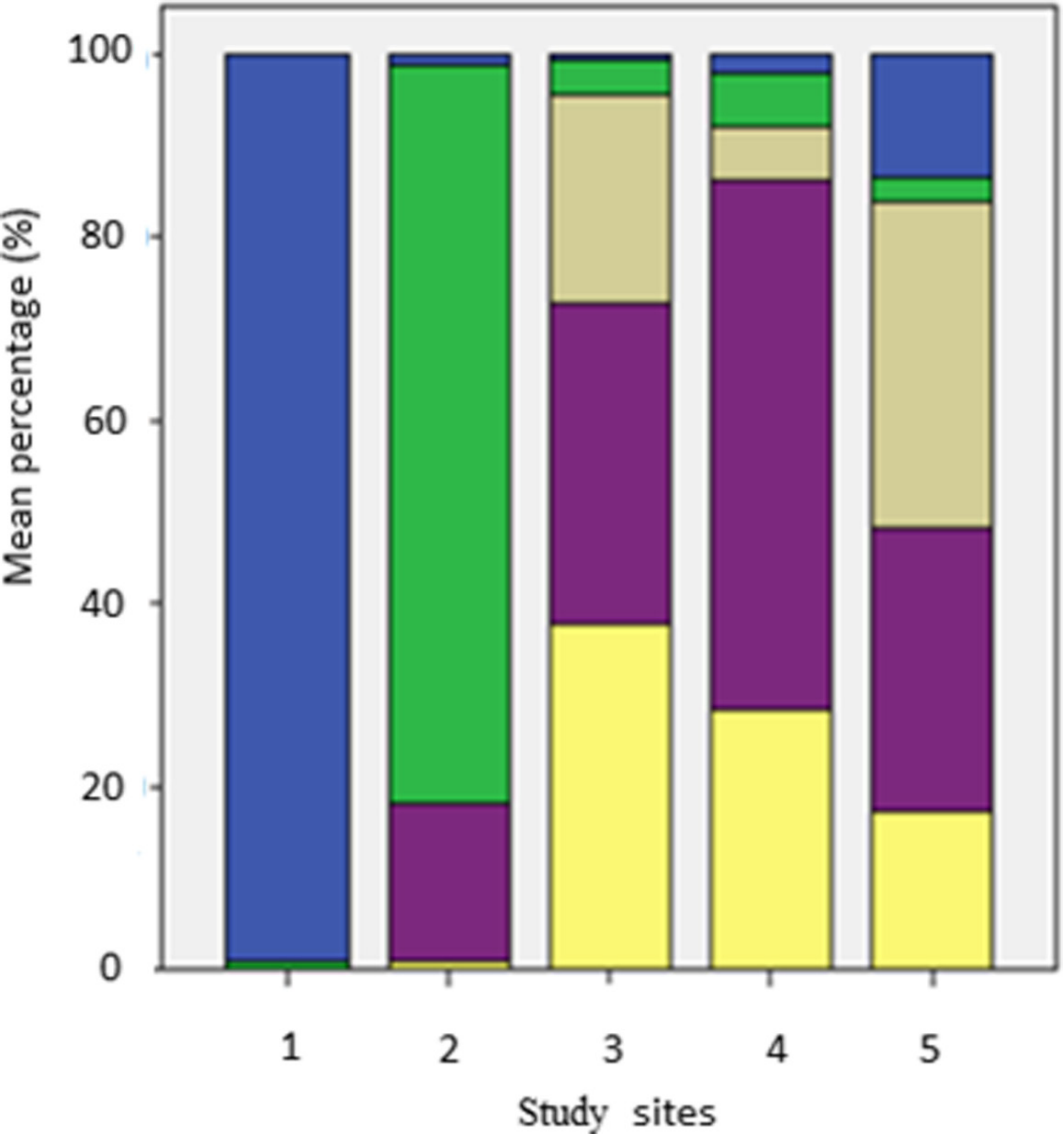
Bar graph of mean percentages of AOM types by sites (1 Snowy River, 2 Club Lake Creek, 3 Diggers Creek, 4 Thredbo River, 5 Mowamba River) across all sampling episodes (February, May, December 2013). The AOM types were: algae/moss [blue], grass [green], gray [large leaves], purple [small leaves], and twigs/bark/wood [yellow].

Total particulate benthic organic matter content significantly differed (*p* < .001) between sites but did not show significant differences between sampling episodes (*F* = 2.175, *p* = .131) and no significant interaction between these two factors (*F* = 0.828, *p* = .585). Sites below the tree line had higher benthic organic matter (ranging from 2.40 to 28.58 g/m^2^ at site 3) than those above the tree line (ranging from 1.15 to 1.51 g/m^2^ at site 1; Figure 5). Similarly, the types of organic matter were significantly different between sites (*F* = 17.97, *p* < .001) but not between sampling episodes (*F* = 2.18, *p* = .131), and there were no interactions (*F* = 0.83, *p* = .585).

Stark differences were shown in the type of benthic organic content across the tree line. Generally, at sites below the tree line, between 90% and 100% of the particulate benthic organic matter is comprised of dicotyledon plants of terrestrial origin, i.e., large dicotyledons leaves, small dicotyledons leaves, and twigs/bark/wood (Figure 5). At the two sites above the tree line, the particulate organic matter was either mostly benthic algae/moss (90%–100%) at the highest site (Snowy River) or mostly monocot grass (≈80%) in Club Lake Creek and ≈15% small leafed terrestrial dicotyledons (Figure 5). There was no detectable seasonal variability in the type and quantity of organic matter found at each site.

### 3.3 Macroinvertebrates in leaf bags and in benthos

Macroinvertebrate community structure in the benthos was different to that in the coarse mesh leaf bags (Pseudo *F*‐value = 22.0; *p* < .001). Site (Pseudo *F*‐value = 8.2; *p* < .001) and sampling episodes (Pseudo *F*‐value = 4.9; *p* < .001) were also important factors that influenced the differences in macroinvertebrate communities in leaf bags. These differences in the macroinvertebrate communities between the leaf bags and benthic samples mostly reflected functional feeding roles between sites above and below the tree line. For instance, the shredder caddisfly larvae (Helicophidae/Calosidae) and Conoesucidae were roughly four to eight times more abundant below (Diggers Creek and Thredbo River) than above the tree line (Snowy River) in both the coarse mesh bags and in the benthos. In contrast, Gripopterygidae, Baetidae, and Elmidae (collector functional feeding group taxa) were generally more common in the benthos than in the leaf bags and were two to three times higher in streams above than below the tree line (Table S1).

Coarse mesh bags in streams below the tree line attracted a fairly similar number of macroinvertebrate taxa and were dominated mainly by shredders such as Helicophidae/Calosidae and Conoesucidae. The high average dissimilarity (73%) between macroinvertebrates from the coarse mesh bags and benthic samples was due to the higher abundances of the families Leptophlebidae, Gripopterygidae, and Elmidae in benthos relative to coarse mesh bags. SIMPER analysis showed that Gripopterygidae caused the greatest dissimilarity between the benthic and coarse mesh bag samples (10.1%); being four times more abundant in the benthos than in the coarse mesh bags (Table S1).

## 4 DISCUSSION

Our results suggest that warmer stream water from both seasonal and elevational changes enhanced leaf litter breakdown through both microbial and macroinvertebrate activities. Macroinvertebrates and microbes equally contributed to the breakdown of leaf litter.

### 4.1 Break‐down of leaf litter and cotton‐strips

The relationships found between higher leaf litter breakdown rates at warmer water temperatures highlight the influence of elevation and seasons on nutrient processing. ANOVA showed some differences in the breakdown rates of leaves and cotton strips between sites for both coarse and fine mesh bags. However, when the linear effect of temperature during the deployment was taken into account, there was no evidence of any differences between sites (Figure 2), suggesting that all differences in breakdown rates (whether for leaves or cotton strips and in coarse or fine mesh bags) between sites, could be accounted for by temperature.

The rates of leaf litter breakdown (per day) were consistently greater in the coarse mesh bags relative to that in the fine meshed bag. This result supports the involvement of macroinvertebrates in leaf litter breakdown rates independent of changes over different sampling episodes. Our results also suggest that the involvement of both microbial and macroinvertebrate communities are equally important in litter breakdown across water temperatures. Other researchers have related increased leaf litter breakdown rates with decreasing elevation (Liu et al., 2017) and shown shredder‐mediated leaf litter breakdown rates increased with elevation, when normalized for degree days and N concentrations (Jinggut & Yule, 2015). In contrast, our study found increased macroinvertebrate activity below the tree line relative to that above the tree line, possibly explained by the increased presence of shredder invertebrate taxa. Nevertheless, temperature alone explained the increase in litter breakdown rates at the lower elevation sites.

The rate of cotton tensile loss (*k*‐value per day) was less variable when deployed for a shorter duration (28 days). However, it was highly variable over longer deployment periods (e.g., June–October). Cotton tensile loss was positively related to the overall period of deployment in streams, as seen elsewhere (e.g., Piggott et al., 2015; Tiegs et al., 2013, 2019). Piggott et al. (2015) showed that cotton strips exposed for 7 days while water temperatures were raised had a positive but weakly unimodal effect on tensile strength. The differences in water temperatures between different deployment periods in our study may influence microbial community structure affecting the overall enzymatic capacity to breakdown important structural polysaccharides in the cotton strips (Piggott et al., 2015).

Rates of cotton tensile loss and leaf litter breakdown were generally consistent between deployments and sites. For example, during warmer periods, both the rates of cottons tensile loss and the leaf litter mass loss were greater than in cooler deployment periods. Rates of tensile loss in cotton strips and mass loss in leaf bags were generally greater below than above the tree line, although they were variable between sites. This suggests that the rate of tensile loss in cotton strips is a useful variable in studying leaf litter breakdown in streams, as shown elsewhere (e.g., Peralta‐Maraver et al., 2019; Schäfer et al., 2012; Tiegs et al., 2019). Cotton strip assays are a potentially relevant proxy for organic matter decomposition studies as they are simple to perform, allowing for high replication; easier to cut to a standard size for comparison between sites and deployment episodes (Obbard & Jones, 1993; Slocum et al., 2009). Tensile loss in cotton strips has been used as an alternative or an additional variable to leaf litter breakdown studies in streams (e.g., Schäfer et al., 2012; Tiegs et al., 2019) and in soils (e.g., Harrison et al., 1988; Slocum et al., 2009). However, arguments persist as to the usefulness of cotton strips and their comparability with leaf litter breakdown. Cotton strips may not be comparable to actual plant polymers, which contain pectins, hemicelluloses, and lignins, yet, it is suitable for determining comparative rates of microbial decomposition (Howard, 1988).

### 4.2 Microbial (fungal) colonization and activity on leaf litter

Ergosterol concentrations varied significantly (*p* < .001) among sampling episodes being generally higher in the warmer deployment periods (Figure S3). This is consistent with fungal colonization and activity on leaf litter promoted by higher water temperatures (Ferreira & Chauvet, 2010). Warmer water temperatures (between 20 and 25°C) have also been shown to increase fungal biomass and activity on leaf litter (Dang et al., 2009). Interestingly, it was only in the fine bags that ergosterol concentration was inversely related to temperature, when in coarse bags, this relationship was positive.

**The ergosterol concentrations were consistently lower in fine mesh bags than in coarse bags. The difference in ergosterol concentrations between coarse and fine mesh bags questions a central assumption of this widely used method that microbial communities, including the amount of fungi, are comparable among both (e.g., Lecerf et al., 2005; Schäfer et al., 2012). Moreover, the invertebrate‐mediated breakdown is calculated by subtracting the leaf mass loss measured in fine mesh bags from that in coarse mesh bags. It is suggestive, though, that the smaller mesh size of 50 μm that excludes invertebrate activity may have effectively reduced fungal colonization and activity as conidia of some fungal species exceed the mesh size. Our findings suggest that the use of bags with different mesh sizes to partition the relative breakdown by microorganisms and invertebrates may be dependent on the mesh sizes employed, and this issue requires further attention.

### 4.3 Macroinvertebrates associated with leaf litter

Macroinvertebrates that belong to the shredder functional feeding group are involved significantly in leaf litter breakdown in streams (Merritt et al., 2002). The dominance of caddisfly shredder taxa (Conoesucidae, Helicophidae/Calosidae, and Odontoceridae) in the leaf bag colonization study suggests major involvement in leaf litter breakdown. Higher densities of benthic shredder taxa observed below the tree line than above is likely reflective of a greater abundance in food resources, including allochthonous organic matter. Some shredders were dispersing wider than their normal food source range (leaf litter from trees) and were aggregating in the leaf bags, which represents an island of the abundance of palatable food. For instance, Helicophidae/Calosidae was found at larger densities in the benthos as well as in the leaf bags both above and below the tree line. In contrast, Conoesucidae, a shredder reported elsewhere in the southern hemisphere (Miserendino & Pizzolon, 2003; Winterbourn et al., 2008), was observed at relatively lower densities in the leaf bags and in benthic samples. These trends in the distribution of benthic shredder taxa support suggestions of the influence of elevational gradients (Camacho et al., 2009), temperature regimes (Salmah et al., 2013), and riparian vegetation (Claeson et al., 2014; Masese et al., 2014).

### 4.4 Input of organic matter into the streams

Streams above the tree line tended to have a higher representation of benthic algae than streams below it, with shading by trees being a potential limiting factor in autochthonous production (Figure 5). A generally reduced input of organic matter at sites above the tree line was evident from the present study. We hypothesize that the relative increase of organic matter at those sites during warmer periods indicates reduced flushing due to low discharge, water velocity, and depth.

There was less diversity in the organic matter above than below the tree line. This is likely a reflection of differences in riparian and adjacent vegetation types; rocky or barren with occasional alpine herbs and grass above the tree line but well vegetated below it (Table 1; Costin, 1989; Lecerf et al., 2005). An assortment of organic matter below the tree line associated with local riparian vegetation of mainly *Eucalyptus* spp. in streams below the tree line was found.

Temperature is an important factor in the process of the breakdown of organic matter. Further positive changes in air and, thus, water temperature regimes associated with climate change would see increased breakdown rates of organic matter in streams by microbial and macroinvertebrate activity. This is supported by the increased leaf litter breakdown rate associated with warmer periods of the year and at lower elevations (see Table 3; Figure 2). There may also be an upward shift in the tree line boundary like those reported elsewhere (e.g., Gatti et al., 2019; Leonelli et al., 2011). If these changes in vegetation occur, greater abundances of shredder macroinvertebrates that generally associate with organic litter as their food resources may proliferate, which in turn will result in the greater breakdown of organic matter by macroinvertebrates.

## 5 CONCLUSION

Increases in water temperatures at lower elevations and during warmer periods of the year enhanced the rate of leaf litter and cotton strip breakdown. Microbial and shredder macroinvertebrate activities on leaf litter were highly influenced by spatio‐temporal variations in stream temperature and spatial availability of organic matter. Climate–change–induced temperature increases in streams may potentially increase nutrient processing through increased rates of the breakdown of organic matter.

## AUTHOR CONTRIBUTIONS

**Lloyd P. Werry:** Conceptualization (supporting); data curation (lead); formal analysis (equal); investigation (lead); methodology (equal); project administration (supporting); visualization (supporting); writing – original draft (lead); writing – review and editing (lead). **Mirco Bundschuh:** Formal analysis (equal); investigation (equal); resources (supporting); validation (equal); writing – review and editing (equal). **Simon M. Mitrovic:** Formal analysis (equal); investigation (supporting); project administration (equal); resources (supporting); software (equal); supervision (equal); validation (equal); writing – review and editing (equal). **Richard Lim:** Formal analysis (supporting); investigation (supporting); project administration (supporting); resources (equal); supervision (equal); validation (equal); visualization (supporting); writing – review and editing (equal). **Ben J. Kefford:** Conceptualization (lead); data curation (equal); formal analysis (equal); funding acquisition (lead); investigation (supporting); methodology (supporting); project administration (lead); resources (supporting); software (supporting); supervision (lead); validation (equal); visualization (equal); writing – review and editing (equal).

## FUNDING INFORMATION

The work was supported by Australian Research Council Discovery Project (DP180102016) through Ben J Kefford.

## CONFLICT OF INTEREST

We declare that there are no conflicts of interest among persons involved in the preparation of this manuscript.

## Supporting information


Appendix S1

## Data Availability

The data that support the findings of this study are openly available in Dryad at https://doi.org/10.5061/dryad.tdz08kq2n.

## References

[ece39433-bib-0001] Andersons, M. J. (2001). A new methods for non‐parametric multivariate analysis of variance. Austral Ecology, 26(1), 32–46.

[ece39433-bib-0002] Benfield, E. F. , Fritz, K. M. , & Tiegs, S. D. (2017). Leaf‐litter breakdown. In G. A. Lamberti & F. R. Hauer (Eds.), Methods in stream ecology (3rd ed., pp. 71–82). Academic Press. 10.1016/B978-0-12-813047-6.00005-X

[ece39433-bib-0003] Bo, T. , Cammarata, M. , Lopez‐Rodrigues, M. J. , De Figueroa, J. M. T. , & Fenoglio, S. (2014). Leaf litter decomposition and invertebrate colonization in alpine environments above the tree line: An experimental study. Polish Journal of Ecology, 62(2), 217–225.

[ece39433-bib-0004] Boulton, A. J. , & Boon, P. I. (1991). A review of methodology used to measure leaf litter decomposition in lotic environments: Time to turn over an old leaf. Australian Journal of Marine and Freshwater Research, 42, 1–43.

[ece39433-bib-0005] Camacho, R. , Boyero, L. , Cornejo, A. , & Ibanez, A. (2009). Local variation in shredder distribution can explain their oversight in tropical streams. Biotropica, 41(5), 625–632. 10.1111/j.1744-7424.2009.00519.x

[ece39433-bib-0006] Claeson, S. M. , LeRoy, C. J. , Barry, J. R. , & Kuehn, K. A. (2014). Impacts of invasive riparian knotweed on litter decomposition, aquatic fungi, and macroinvertebrates. Biological Invasions, 16, 1531–1544.

[ece39433-bib-0007] Clark, K. R. , & Warwick, R. M. (2001). Changes in marine communities: An approach to statistical analysis and interpretation (2nd ed.). PRIMER‐E, Ltd., Plymouth Marine Laboratory.

[ece39433-bib-0008] Costin, A. B. (1989). The Alps in the global perspectives. In R. B. Good (Ed.), The scientific significance of the Australian Alps (pp. 7–21). Australian Alps Liaison Committee.

[ece39433-bib-0009] Costin, A. B. , Gray, M. , Totterdell, C. J. , & Wimbush, D. J. (2000). Kosciuszko Alpine Flora. CSIRO Publishing. 10.1071/9780643101142

[ece39433-bib-0010] Dang, C. K. , Schindler, M. , Chauvet, E. , & Gessner, M. O. (2009). Temperature oscillation coupled with fungal community shifts can modulate warming effects on litter decomposition. Ecology, 90(1), 122–131. 19294919 10.1890/07-1974.1

[ece39433-bib-0011] Dobson, M. , & Hildrew, A. G. (1992). A test of resource limitation among shredding detritivores in low order streams in southern England. Journal of Animal Ecology, 61, 69–71.

[ece39433-bib-0012] Ferreira, V. , & Chauvet, E. (2010). Synergistic effects of water temperature and dissolved nutrients on litter decomposition and associated fungi. Global Change Biology, 17(1), 551–564.

[ece39433-bib-0013] Gatti, R. C. , Callaghan, T. , Velichevskaya, A. , Dudko, A. , Fabbio, L. , Battipalgia, G. , & Liang, J. (2019). Accelerating upward tree line shift in the Altai Mountains under last century climate change. Scientific Reports, 9, 7678. 10.1038/s41598-019-44188-1 31118471 PMC6531548

[ece39433-bib-0014] Gessner, M. O. , & Chauvet, E. (1994). Importance of stream micro‐fungi in controlling breakdown rates of leaf litter. Ecology, 75, 1807–1817.

[ece39433-bib-0015] Gessner, M. O. , & Chauvet, E. (2002). A case for using litter breakdown to assess functional stream integrity. Ecological Applications, 12, 498–510.

[ece39433-bib-0016] Gessner, M. O. , & Schmitt, A. L. (1996). Use of solid‐phase extraction to determine ergosterol concentration in plant tissue colonized by fungi. Applied Environmental Microbiology, 62(2), 415–419. 16535229 10.1128/aem.62.2.415-419.1996 PMC1388767

[ece39433-bib-0017] Gill, B. A. , Kondratieff, B. C. , Casner, K. L. , Encalada, A. C. , Flecker, A. S. , Gannon, D. G. , Ghalambor, C. K. , Guayasamin, J. M. , Poff, N. L. , Simmons, M. P. , Thomas, S. A. , Zamudio, K. R. , & Funk, W. C. (2016). Cryptic species diversity reveals biogeographic support for the ‘mountain passes are higher in the tropics’ hypothesis. Proceedings of the Royal Society B, 283, 20160553. 10.1098/rspb.2016.0553 27306051 PMC4920318

[ece39433-bib-0018] Harrison, A. F. , Latter, P. M. , & Walton, D. W. H. (1988). Cotton strip assay: An index of decomposition in soils. Institute of Terrestrial Ecology.

[ece39433-bib-0019] Hennessy, K. , Fitzharris, B. , Bates, B. C. , Harvey, N. , Howden, S. M. , Salinger, J. , & Warrick, R. (2007). Australia and New Zealand. In M. L. Parry , O. F. Canziani , J. P. Palutikof , P. J. V. D. Linden , & C. E. Hanson (Eds.), Climate Change 2007: Impacts, Adaptation and Vulnerability. Contribution of Working Group II to the Fourth Assessment Report of the Intergovernmental Panel on Climate Change (pp. 507–540). Cambridge University Press.

[ece39433-bib-0020] Hieber, M. , & Gessner, M. O. (2002). Contribution of stream detritivores, fungi, and bacteria to leaf breakdown based on biomass estimates. Ecology, 83, 1026–1038.

[ece39433-bib-0021] Hoover, T. M. , Richardson, J. S. , & Yonemitsu, N. (2006). Flow‐substrate interactions create and mediate leaf litter resource patches in streams. Freshwater Biology, 51(3), 435–447. 10.1111/j.1365-2427.2005.01499.x

[ece39433-bib-0022] Howard, P. J. A. (1988). A critical evaluation of the cotton strip assay. In A. F. Harrison , P. M. Latter , & D. W. H. Walton (Eds.), Cotton strip assay: An index of decomposition in soils (pp. 34–42). Institute of Ecology, Grange‐Over‐Sands.

[ece39433-bib-0023] Humphries, P. , Keckeis, H. , & Finlayson, B. (2014). The river wave concept: Integrating river ecosystem models. Bioscience, 64(10), 870–882. 10.1093/biosci/biu130

[ece39433-bib-0024] Imberger, S. J. , Walsh, C. J. , & Grace, M. R. (2008). More microbial activity, not abrasive flow or shredder abundance, accelerates breakdown of labile leaf litter in urban streams. Journal of the North American Benthological Society, 27, 549–561.

[ece39433-bib-0025] Jinggut, T. , & Yule, C. M. (2015). Leaf litter breakdown in streams of East Malaysia (Borneo) along an altitudinal gradient: Initial nitrogen content of litter limits shredder feeding. Freshwater Science, 34(2), 691–701.

[ece39433-bib-0026] Lecerf, A. , & Chauvet, E. (2008). Diversity and functions of leaf‐decaying fungi in human‐altered streams. Freshwater Biology, 53(8), 1658–1672. 10.11111/j.1365-2427.2008.01986.x

[ece39433-bib-0027] Lecerf, A. , Dobson, M. , Dang, C. K. , & Chauvet, E. (2005). Riparian plant species loss alters trophic dynamics in detritus‐based stream ecosystems. Oecologia, 146(3), 432–442. 16096846 10.1007/s00442-005-0212-3

[ece39433-bib-0028] Leonelli, G. , Pelfini, M. , di Cella, U. M. , & Garavaglia, V. (2011). Climate warming and the recent tree line shift in the European Alps: The role of geomorphological factors in high‐altitude sites. Ambio, 40(3), 264–273. 10.1007/s13280-010-0096-2 21644455 PMC3357808

[ece39433-bib-0029] Ligeiro, R. , Melo, A. S. , & Callisto, M. (2010). Spatial scale and the diversity of macroinvertebrates in a neotropical catchment. Freshwater Biology, 55(2), 424–435. 10.1111/j.1365-2427.2009.02291.x

[ece39433-bib-0030] Liu, G. , Sun, J. , Tian, K. , Yuan, X. , An, S. , & Wang, H. (2017). Litter decomposition of emergent plants along an elevational gradient in wetlands of Yunnan Plateau, China. Chinese Geographical Sciences, 27(5), 760–771.

[ece39433-bib-0031] Manning, D. W. P. , Ferreira, V. , Gulis, V. , & Rosemond, A. D. (2021). Pathways, mechanisms, and consequences of nutrient‐stimulated plant litter decomposition in streams. In C. M. Swan , L. Boyero , & C. Canhoto (Eds.), The ecology of plant litter decomposition in stream ecosystems. Springer. 10.1007/978-3-030-72854-0_16

[ece39433-bib-0032] Marchant, R. , Kefford, B. J. , Wasley, J. , King, C. K. , Doube, J. , & Nugegoda, D. (2011). Response of stream invertebrate communities to vegetation damage from overgrazing by exotic rabbits on subantarctic Macquarie Island. Marine & Freshwater Research, 62, 404–413.

[ece39433-bib-0033] Masese, F. O. , Kitaka, N. , Kipkemboi, J. , Gettel, G. M. , Irvine, K. , & McClain, M. E. (2014). Macroinvertebrate functional feeding groups in Kenyan highland streams: Evidence for a diverse shredder guild. Freshwater Science, 33(2), 435–450. 10.1086/675681

[ece39433-bib-0034] Merritt, R. W. , Cummins, K. W. , Berg, M. B. , Novak, J. A. , Higgins, M. J. , Wessell, K. J. , & Lessard, J. L. (2002). Development and application of a macroinvertebrate functional‐group approach in the bioassessment of remnant river oxbows in southwest Florida. Journal of the North American Benthological Society, 21(2), 290–310.

[ece39433-bib-0035] Miserendino, M. L. , & Pizzolon, L. A. (2003). Distribution of macroinvertebrate assemblages in the Azul‐Quemquemtreu river basin, Patagonia, Argentina. New Zealand Journal of Marine and Freshwater Research, 37, 525–539.

[ece39433-bib-0036] Obbard, J. P. , & Jones, K. C. (1993). The use of the cotton‐strip assay to assess cellulose decomposition in heavy metal‐contaminated sewage sludge‐amended soils. Environmental Pollution, 81(2), 173–178. 15091827 10.1016/0269-7491(93)90083-z

[ece39433-bib-0037] Peralta‐Maraver, I. , Perkins, D. M. , Thompson, M. S. A. , Fussmann, K. , Reiss, J. , & Robertson, A. L. (2019). Comparing biotic drivers of litter breakdown across stream compartments. The Journal of Animal Ecology, 88(8), 1146–1157. 10.1111/1365-2656.13000 31032898 PMC6851634

[ece39433-bib-0038] Pickering, C. (2007). Climate change and other threats in the Australian Alps. In M. Taylor & P. Figgis (Eds.), Protected Areas: Buffering nature against climate change. Proceedings of a WWF and IUCN World Commission on Protected Areas symposium, 18–19 June 2007, Canberra (pp. 28–34). WWF‐Australia.

[ece39433-bib-0039] Piggott, J. J. , Niyogi, D. K. , Townsend, C. R. , & Matthaei, C. D. (2015). Multiple stressors and stream ecosystem functioning: Climate warming and agricultural stressors interact to affect processing of organic matter. Journal of Applied Ecology, 52(5), 1126–1134.

[ece39433-bib-0040] Salinas, N. , Malhi, Y. , Meir, P. , Silman, M. , Roman Cuesta, R. , Huaman, J. , Salinas, D. , Huaman, V. , Gibaja, A. , Mamani, M. , & Farfan, F. (2011). The sensitivity of tropical leaf litter decomposition to temperature: Results from a large‐scale leaf translocation experiment along an elevation gradient in Peruvian forests. New Phytologist, 189(4), 967–977. 10.1111/j.1469-8137.2010.03521.x 21077887

[ece39433-bib-0041] Salmah, M. R. C. , Al‐Shami, S. A. , Hassan, A. A. , Madrus, M. R. , & Huda, A. N. (2013). Distribution of detritivores in tropical forest streams of peninsular Malaysia: Role of temperature, canopy cover and altitude variability. International Journal of Meteorology, 58(5), 679–690. 10.1007/s00484-013-0648-9 23483291

[ece39433-bib-0042] Schäfer, R. B. , Bundschuh, M. , Rouch, D. A. , Szöcs, E. , von der Ohe, P. C. , Pettigrove, V. J. , Schulz, R. , Nugegoda, D. , & Kefford, B. J. (2012). Relationships of selected ecosystem functions in streams pesticide toxicity, salinity, and other environmental variables on selected ecosystem functions in streams and the relevance for ecosystem services. Science of the Total Environment, 415, 69–78. 21802709 10.1016/j.scitotenv.2011.05.063

[ece39433-bib-0043] Shah, A. A. , Gill, B. A. , Encalada, A. C. , Flecker, A. S. , Funk, W. C. , Guayasamin, J. M. , Kondratieff, B. C. , Poff, N. L. , Thomas, S. A. , Zamudio, K. R. , & Ghalambor, C. K. (2017). Climate variability predicts thermal limits of aquatic insects across elevation and latitude. Functional Ecology, 31, 2118–2127. 10.1111/1365-2435.12906

[ece39433-bib-0044] Shah, R. D. T. , Sharma, S. , Peter, H. , Janhig, S. C. , & Pauls, S. U. (2015). The climate sensitive zone along an altitudinal gradient in central Himalayan rivers: A useful concept to monitor climate change impacts in mountain regions. Climatic Change, 132(2), 265–278.

[ece39433-bib-0045] Slocum, M. G. , Roberts, J. , & Mendelssohn, I. A. (2009). Artist canvas as a new standard for the cotton strip assay. Journal of Plant Nutrition and Soil Science, 172, 71–74.

[ece39433-bib-0046] Suberkropp, K. , & Chauvet, E. (1995). Regulation of breakdown by fungi in streams: Influences of water chemistry. Ecology, 76, 1433–1445.

[ece39433-bib-0047] Suter, P. , Clair, S. R. , Hawking, J. , & Bryce, C. (2002). Aquatic macroinvertebrates from streams in the Mt Kosciuszko area (pp. 90–97). Biodiversity in the Snowy Mountains. G. K. Australian Institute of Alpine Studies.

[ece39433-bib-0048] Tank, J. L. , Rosi‐Marshall, E. J. , Griffiths, N. A. , Entrekin, S. A. , & Stephen, M. L. (2010). A review of allochthonous organic matter dynamics and metabolism in streams. Journal of North American Benthological Society, 29(1), 118–146.

[ece39433-bib-0049] Taylor, B. R. , & Andrushchenko, I. V. (2014). Interaction of water temperature and shredders on leaf litter breakdown: A comparison of streams in Canada and Norway. Hydrobiologia, 721, 77–88. 10.1007/s10750-013-1650-2

[ece39433-bib-0050] Tiegs, S. D. , Clapcott, J. E. , Griffiths, N. A. , & Boulton, A. J. (2013). A standard cotton‐strip assay for measuring organic‐matter decomposition in streams. Ecological Indicators, 32, 131–139.

[ece39433-bib-0051] Tiegs, S. D. , Costello, D. M. , Isken, M. W. , Woodward, G. , McIntyre, P. B. , Gessner, M. O. , Chauvet, E. , Griffiths, N. A. , Flecker, A. S. , Acuña, V. , Albariño, R. , Allen, D. C. , Alonso, C. , Andino, P. , Arango, C. , Aroviita, J. , Barbosa, M. V. M. , Barmuta, L. A. , Baxter, C. V. , … Zwart, J. A. (2019). Global patterns and drivers of ecosystem functioning in rivers and riparian zones. Science Advance, 5, eaav0486. 10.1126/sciadv.aav0486 PMC6326750 30662951

[ece39433-bib-0052] Tilman, D. (1982). Resource competition and community structure. Monogr. Pop. Biol. 17. Princeton University Press 296 p. 7162524

[ece39433-bib-0053] Vannote, R. L. , Minshall, G. W. , Cummins, K. W. , Sedell, J. R. , & Cushing, C. E. (1980). The river continuum concept. Canadian Journal of Fisheries and Aquatic Science, 37, 130–137.

[ece39433-bib-0054] Wahizatul, A. A. , Long, S. H. , & Ahmad, A. (2011). Composition and distribution of aquatic insect communities in relation to water quality in two freshwater streams of Hulu Terengganu, Terengganu. Journal of Sustainability Science and Management, 6(1), 148–155.

[ece39433-bib-0055] Wang, X. , Liu, X. , & Hurren, C. (2008). Physical and mechanical testing of textiles. In J. Hu (Ed.), Fabric testing (pp. 90–124). Woodhead Publishing Ltd.

[ece39433-bib-0056] Ward, J. V. (1992). Aquatic Insect Ecology. 1. Biology and Habitat. John Wiley & Sons, Inc.

[ece39433-bib-0057] Winterbourn, M. J. , Cadbury, S. , Ilg, C. , & Milner, A. M. (2008). Mayfly production in a New Zealand glacial stream and the potential effect of climate change. Hydrobiologia, 603, 211–219.

[ece39433-bib-0058] Woodward, G. , Gessner, M. , Giller, P. , Gulis, V. , Hladys, S. , Lecerf, A. , Malmqvist, B. , Mackie, B. , Tiegs, S. D. , Cariss, H. , Dobson, M. , Elosegi, A. , Ferreira, V. , Graca, M. , Fleituch, T. , Lacoursiere, J. , Nistorescu, M. , Pozo, J. , Risnoveanu, G. , … Chauvet, E. (2012). Continental‐scale effects of nutrient pollution on stream ecosystem functioning. Science, 336, 1430–1440. 10.1126/science.1219534 22700929

[ece39433-bib-0059] Young, R. G. , Matthaei, C. D. , & Townsend, C. R. (2008). Organic matter breakdown and ecosystem metabolism: Functional indicators for assessing river ecosystem health. Journal of the North American Benthological Society, 27, 605–625.

[ece39433-bib-0060] Zhai, J. , Anderson, J. T. , Yan, G. , Cong, L. , Wu, Y. , Dai, L. , Liu, J. , & Zhang, Z. (2021). Decomposition and nutrient dynamics responses of plant litter to interactive effects of flooding and salinity in Yellow River Delta wetland in north‐eastern China. Ecological Indicators, 120, 106943. 10.1016/j.ecolind.2020.106943

