# Supplementary material for: Leaf litter breakdown along an elevational gradient in Australian alpine streams
Source: Ecol Evol. 2022 Oct 18;12(10):e9433. doi: 10.1002/ece3.9433 (PMC9596332; doi:10.1002/ece3.9433)
Supplement: Supplementary file 1 — Appendix S1 [file ECE3-12-e9433-s001.docx]

**Supplementary Table S1.** Summary of SIMPER results for macroinvertebrates families on leaf bags and Surber samples across all sampling episodes between streams above and below the tree-line: average abundance (% cover) of discriminating families between sampling methods and between streams above and below the tree-line, their contribution (%) to the dissimilarity between groups and cumulative total (%) of contributions. Average abundance (%) of families in ‘shredder’ functional group are in BOLD.

| **FAMILY** | **AVERAGE ABUNDANCE** | | **AVERAGE DISSIMILARITY** | **CONTRIBUTION** | **CUMULATIVE CONTRIBUTION** |
| --- | --- | --- | --- | --- | --- |
|  | **LEAF BAGS** | **SURBER SAMPLES** |  |  |  |
|  |  |  | ***72.8%*** |  |  |
| Gripopterygidae | 1.02 | 4.52 | 10.1 | 14.2 | 14.2 |
| Leptophlebidae | 1.67 | 4.55 | 9.48 | 13.0 | 27.2 |
| Helicophidae/Calosidae | **3.37** | **1.39** | 5.88 | 8.08 | 35.3 |
| Elmidae | 0.54 | 2.43 | 5.51 | 7.57 | 42.9 |
|  |  |  |  |  |  |
|  | **ON LEAF** | **BAGS ONLY** |  |  |  |
|  | ABOVE TREE LINE | BELOW TREE LINE |  |  |  |
|  | ***Snowy River*** | ***Diggers Creek*** | ***68%*** |  |  |
| Conoesucidae | **0.31** | **8.14** | 14.6 | 21.4 | 21.4 |
| Gripopterygidae | 4.09 | 4.12 | 6.45 | 9.47 | 30.8 |
| Leptophlebidae | 3.28 | 3.96 | 6.34 | 9.32 | 40.2 |
| Helicophidae/Calosidae | **0.89** | **3.50** | 5.60 | 8.23 | 48.4 |
|  | ***Snowy River*** | ***Thredbo River*** | ***64.8 %*** |  |  |
| Conoesucidae | **0.31** | **3.19** | 6.79 | 10.5 | 10.5 |
| Gripopterygidae | 4.09 | 2.37 | 6.76 | 10.4 | 20.4 |
| Leptophlebidae | 3.28 | 2.84 | 5.07 | 7.83 | 28.7 |
| Helicophidae/Calosidae | **0.89** | **2.68** | 5.06 | 7.81 | 36.6 |
|  | ***Snowy River*** | ***Mowamba River*** | ***64.3 %*** |  |  |
| Helicophidae/Calosidae | **0.89** | **3.04** | 6.39 | 9.93 | 9.93 |
| Gripopterygidae | 4.09 | 1.97 | 5.92 | 9.20 | 19.1 |
| Leptophlebidae | 3.28 | 3.84 | 5.62 | 8.74 | 27.9 |

|  | ***Club Lake Creek*** | ***Thredbo River*** | ***63.9 %*** |  |  |  |
| --- | --- | --- | --- | --- | --- | --- |
| Gripopterygidae | 5.12 | 2.37 | 8.57 | 1.44 | 13.4 | 13.4 |
| Leptophlebidae | 4.94 | 2.84 | 6.98 | 1.20 | 10.9 | 24.1 |
| Scirtidae | 3.00 | 0.20 | 6.15 | 1.40 | 9.63 | 34.0 |
| Helicophidae/Calosidae | **0.23** | **2.68** | 5.65 | 0.75 | 8.85 | 42.8 |
|  | ***Club Lake Creek*** | ***Diggers Creek*** | ***62.0 %*** |  |  |  |
| Conoesucidae | **1.65** | **8.14** | 12.0 | 2.23 | 19.4 | 19.4 |
| Leptophlebidae | 4.94 | 3.96 | 7.65 | 1.16 | 12.3 | 31.7 |
| Gripopterygidae | 5.12 | 4.12 | 7.00 | 1.16 | 11.3 | 43.0 |
| Helicophidae/Calosidae | **0.23** | **3.50** | 5.24 | 0.74 | 8.45 | 51.4 |
|  | ***Club Lake Creek*** | ***Mowamba River*** | ***61.6 %*** |  |  |  |
| Gripopterygidae | 5.12 | 1.97 | 7.37 | 1.14 | 12.0 | 12.0 |
| Helicophidae/Calosidae | **0.23** | **3.04** | 6.70 | 0.70 | 10.9 | 29.7 |
| Leptophlebidae | 4.94 | 3.84 | 6.41 | 1.14 | 10.4 | 13.3 |


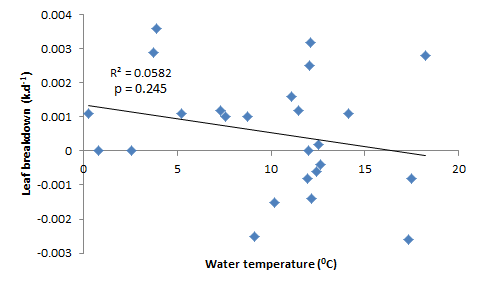


**Supplementary Figure S2**. Rate of leaf litter breakdown (*k*.d^-1^) due to macroinvertebrate activity with increasing temperature. An inverse relationship of R2 = 0.0582 (p = 0.245).


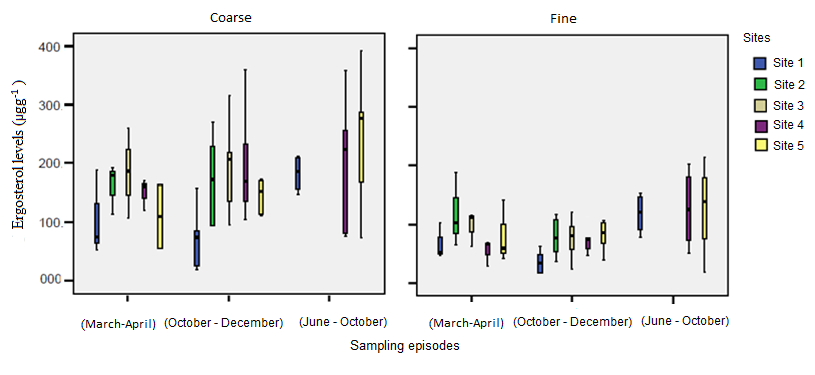


**Supplementary Figure S3**. Boxplot showing ergosterol levels (ug.g^-1^) in coarse and fine leaf packs placed in five alpine streams in the upper Snowy River catchment over March-April, June-October, and October-December periods of 2013. (Sites: 1 Snowy River, 2 Club Lake Creek, 3 Diggers Creek, 4 Thredbo River, Mowamba River). Lines in the plot indicate level of variability in data.
